# Supplementary material for: A fault-intrusion-tolerant system and deadline-aware algorithm for scheduling scientific workflow in the cloud
Source: PeerJ Comput Sci. 2021 Nov 2;7:e747. doi: 10.7717/peerj-cs.747 (PMC8576554; doi:10.7717/peerj-cs.747)
Supplement: Supplemental Information 6 [file peerj-cs-07-747-s006.zip › Myproject1.1/docs/org/cloudbus/cloudsim/lists/HostList.html]

HostList


JavaScript is disabled on your browser.


- Overview
- Package
- Class
- Use
- Tree
- Deprecated
- Index
- Help

- Prev Class
- Next Class

- Frames
- No Frames

- All Classes

- Summary:
- Nested |
- Field |
- Constr |
- Method

- Detail:
- Field |
- Constr |
- Method


org.cloudbus.cloudsim.lists

## Class HostList

- java.lang.Object
- - org.cloudbus.cloudsim.lists.HostList

- ---

    

  ```
  public class HostList
  extends java.lang.Object
  ```

  HostList is a collection of operations on lists of hosts.

  Since:
  :   CloudSim Toolkit 2.0

- - ### Constructor Summary

    Constructors

    Constructor and Description

    `HostList()`
  - ### Method Summary

    Methods

    | Modifier and Type | Method and Description |
    | --- | --- |
    | `static <T extends Host>  T` | `getById(java.util.List<T> hostList, int id)` Gets the Machine object for a particular ID. |
    | `static <T extends Host>  T` | `getHostWithFreePe(java.util.List<T> hostList)` Gets a Machine with free Pe. |
    | `static <T extends Host>  T` | `getHostWithFreePe(java.util.List<T> hostList, int pesNumber)` Gets a Machine with a specified number of free Pe. |
    | `static <T extends Host>  int` | `getNumberOfBusyPes(java.util.List<T> hostList)` Gets the total number of BUSY PEs for all Machines. |
    | `static <T extends Host>  int` | `getNumberOfFreePes(java.util.List<T> hostList)` Gets the total number of FREE or non-busy PEs for all Machines. |
    | `static <T extends Host>  int` | `getNumberOfPes(java.util.List<T> hostList)` Gets the total number of PEs for all Machines. |
    | `static <T extends Host>  boolean` | `setPeStatus(java.util.List<T> hostList, int status, int hostId, int peId)` Sets the particular Pe status on a Machine. |

    - ### Methods inherited from class java.lang.Object

      `clone, equals, finalize, getClass, hashCode, notify, notifyAll, toString, wait, wait, wait`

- - ### Constructor Detail


    - #### HostList

      ```
      public HostList()
      ```
  - ### Method Detail


    - #### getById

      ```
      public static <T extends Host> T getById(java.util.List<T> hostList,
                               int id)
      ```

      Gets the Machine object for a particular ID.

      Type Parameters:
      :   `T` - the generic type

      Parameters:
      :   `hostList` - the host list
      :   `id` - the host ID

      Returns:
      :   the Machine object or null if no machine exists

      See Also:
      :   `gridsim.Machine`


    - #### getNumberOfPes

      ```
      public static <T extends Host> int getNumberOfPes(java.util.List<T> hostList)
      ```

      Gets the total number of PEs for all Machines.

      Type Parameters:
      :   `T` - the generic type

      Parameters:
      :   `hostList` - the host list

      Returns:
      :   number of PEs


    - #### getNumberOfFreePes

      ```
      public static <T extends Host> int getNumberOfFreePes(java.util.List<T> hostList)
      ```

      Gets the total number of FREE or non-busy PEs for all Machines.

      Type Parameters:
      :   `T` - the generic type

      Parameters:
      :   `hostList` - the host list

      Returns:
      :   number of PEs


    - #### getNumberOfBusyPes

      ```
      public static <T extends Host> int getNumberOfBusyPes(java.util.List<T> hostList)
      ```

      Gets the total number of BUSY PEs for all Machines.

      Type Parameters:
      :   `T` - the generic type

      Parameters:
      :   `hostList` - the host list

      Returns:
      :   number of PEs


    - #### getHostWithFreePe

      ```
      public static <T extends Host> T getHostWithFreePe(java.util.List<T> hostList)
      ```

      Gets a Machine with free Pe.

      Type Parameters:
      :   `T` - the generic type

      Parameters:
      :   `hostList` - the host list

      Returns:
      :   a machine object or null if not found


    - #### getHostWithFreePe

      ```
      public static <T extends Host> T getHostWithFreePe(java.util.List<T> hostList,
                                         int pesNumber)
      ```

      Gets a Machine with a specified number of free Pe.

      Type Parameters:
      :   `T` - the generic type

      Parameters:
      :   `hostList` - the host list
      :   `pesNumber` - the pes number

      Returns:
      :   a machine object or null if not found


    - #### setPeStatus

      ```
      public static <T extends Host> boolean setPeStatus(java.util.List<T> hostList,
                                         int status,
                                         int hostId,
                                         int peId)
      ```

      Sets the particular Pe status on a Machine.

      Type Parameters:
      :   `T` - the generic type

      Parameters:
      :   `hostList` - the host list
      :   `status` - Pe status, either Pe.FREE or Pe.BUSY
      :   `hostId` - the host id
      :   `peId` - the pe id

      Returns:
      :   true if the Pe status has changed, false otherwise (Machine id or
          Pe id might not be exist)


- Overview
- Package
- Class
- Use
- Tree
- Deprecated
- Index
- Help

- Prev Class
- Next Class

- Frames
- No Frames

- All Classes

- Summary:
- Nested |
- Field |
- Constr |
- Method

- Detail:
- Field |
- Constr |
- Method
